# An Econometric Analysis of Climatic Effects on Total Factor Productivity Across U.S. Dairy Counties

**DOI:** 10.3390/ani16010030

**Published:** 2025-12-22

**Authors:** Kamil Bora Bolat, Merve Bolat, Boris E. Bravo-Ureta

**Affiliations:** 1 Department of Agricultural Economics, Field Crops Central Research Institute, Ankara 06170, Türkiye; merveturanbolat@gmail.com; 2 Department of Agricultural Economics, Graduate School of Natural and Applied Sciences, Ankara University, Ankara 06110, Türkiye; 3 Department of Agricultural and Resource Economics, University of Connecticut, Storrs, CT 06269, USA; boris.bravoureta@uconn.edu

**Keywords:** dairy farming, climatic effects, heat stress, stochastic production frontiers, random parameters, total factor productivity

## Abstract

High-yielding dairy cows are particularly vulnerable to heat stress, a challenge that climate change exacerbates. To quantify the impact of climatic variables on productivity, we applied a random parameter stochastic production frontier model to the United States Department of Agriculture (USDA) census data from 1978 to 2022 for 179 dairy counties, allowing us to decompose total factor productivity growth (TFPG). Our analysis indicates that technological advancements were the primary driver of TFPG, amounting to 2.52% annually. While these gains are modestly constrained by heat stress, the average impact on the overall TFPG rate was only 0.008% per year. This minimal impact is consistent with the adoption of strategies such as cooling systems and improved management. Even in the most affected counties, the effect remained slight, with the largest reduction reaching 0.08%. This limited impact suggests that the sector’s adoption of technologies and management strategies appears to have mitigated potential productivity losses. This study highlights that future research is needed to quantify the direct impact of specific on-farm adaptation strategies on dairy productivity to inform well-targeted policy recommendations.

## Simple Summary

High-yielding dairy cows face increasing heat stress due to climate change. Our study analyzed over four decades of data from U.S. dairy counties to understand the economic implications of this challenge. Using a detailed statistical model, we separated the effects of technological progress from climatic pressures on total factor productivity. Our findings show that while technology is the primary driver of productivity growth, heat stress imposes a modest constraint on this growth. Although nationwide results indicate a low reduction in the rate of productivity growth from heat, our analysis reveals that this impact varies by region. More importantly, the slight overall effect suggests that the U.S. dairy industry has been highly effective in managing these climatic challenges through innovation. This finding implies the sector’s resilience and the success of locally implemented strategies in maintaining long-term stability.

## 1. Introduction

The agricultural, food, and related industries constitute the economic bedrock of many states in the U.S., where the dairy sector is a vital component. Contributing about 12% of the world’s milk output and generating annual revenues nearing USD 51 billion, the U.S. dairy sector is a cornerstone of many rural communities, supporting local services such as feed suppliers, veterinarians, and processors [1,2]. However, reliance on specialized, high-yielding breeds (*Holstein cattle*) exposes it to climatic risks [3,4,5,6]. Indeed, recurrent heat stress has already caused 14–35% declines in milk yields, while diminishing surface water availability—especially in the Southwest—is driving up cooling and irrigation costs [7,8]. Moreover, extreme climatic events threaten feed crops [9,10]. The convergence of these factors suggests a challenging financial future, with potential losses for the U.S. dairy industry projected to surpass USD 1.7 billion by 2050 [11]. Underscoring these climate-driven threats are ongoing market volatility and policy shifts, necessitating more localized research aimed at understanding their effects on productivity [12,13]. Robust county-level data would enable both producers and policymakers to invest in adaptive measures that enhance the resilience and sustainability of U.S. dairy production.

Genetic selection in modern dairy farming, while significantly increasing milk yields, has also exacerbated the physiological vulnerability of dairy cows to heat stress due to their narrow thermoneutral zone [4,14,15,16,17,18]. When ambient temperatures and humidity exceed an optimal range, cows must use considerable energy to maintain homeostasis, diverting resources that would otherwise support milk production [7,19].

Researchers often quantify these physiological challenges using the Temperature-Humidity Index (THI), widely recognized as the key metric for assessing heat stress in dairy cattle [20,21]. When the THI exceeds a threshold of 65, physiological stress symptoms begin to manifest and above a THI of 72 severe productivity losses are observed [22,23,24]. Prolonged exposure to high THI levels further reduces feed intake, milk yield, reproductive performance, and milk quality [25,26,27,28]. Ultimately, climate-induced heat stress poses substantial challenges to dairy cow welfare and to the overall industry productivity [5,29,30,31,32,33,34,35].

A growing body of agro-economic studies focuses on the effects of climatic factors on total factor productivity (TFP); however, most consider the agricultural sector as a whole [36]. Few studies go beyond technical efficiency (TE) at the farm level and decompose TFP into constituent components—such as technological progress (TP), scale efficiency change (SEC), TE change, and climatic or environmental effect indices (CEI)—to understand the underlying drivers of TFPG [37,38,39,40,41,42,43,44]. Research spanning various regions, including Australia, several Latin American countries, and the United States, suggests that TFPG is generally positive, with technological progress consistently identified as the primary driver [37,38,39,44,45]. However, there are notable exceptions, including Bravo-Ureta et al. [42], who reported negative TFPG in El Salvador, indicating that technological change combined with climatic conditions can reduce productivity.

Despite its potential importance, the impact of climatic factors on TFP in the dairy sector has received limited attention [36]. A consistent finding across this literature is that models omitting weather variables are rejected in comparative robustness tests, underscoring their explanatory power [46]. Omitting such variables can lead to biased results, as weather-induced output fluctuations may be attributed incorrectly to technical inefficiency, resulting in lower and more volatile TE scores [47,48]. Another common theme is that TFPG is positive, but climatic effects tend to slow down this growth [43,47,48,49]. Researchers have employed a range of variables to quantify climatic effects, yet the literature consistently concludes that climatic stressors reduce productivity growth, a trend projected to intensify as climate change continues [39,50,51,52]. Finally, several studies identify technological progress as the primary engine of TFP change, even as climatic factors work to offset its gains [40,46,53].

Although substantial evidence shows that climatic shocks reduce agricultural output and disrupt dairy cow physiology, the relationship between weather variability and TFP is often limited to broad regional analyses, which obscure local variation, leaving two primary gaps in the dairy productivity literature. First, most studies assess productivity at a regional level, thereby overlooking significant heterogeneity among U.S. counties [47,48,49,54]. Second, while the THI is commonly used as a heat-stress indicator, few studies utilize it to isolate the direct physiological effects of heat. Even fewer distinguish these effects from indirect climatic impacts on feed availability, which are addressed here using growing degree days (GDD) and precipitation.

This study is based on a 45-year (1978–2022) panel of publicly available, county-level data from the USDA National Agricultural Statistics Service (NASS). This extensive period and open accessibility provide a key advantage over typical farm-level analyses, which often utilize shorter periods and data with restricted access. Addressing these gaps, the primary objective of this study is to analyze TFPG across major U.S. dairy counties, with a special focus on the role of climatic factors. Specifically, we aim to assess the effects of alternative definitions of climatic factors on dairy farm productivity over time and space and identify the drivers of TFP by decomposing it into six elements: technological progress; scale efficiency change; technical efficiency change; an environmental index for direct heat stress (THI); an environmental index for indirect climatic impacts on feed ((GDD) and precipitation); and statistical noise. To achieve this, our analysis covers 179 major dairy counties distributed across the Northeast, Midwest, Southeast, and West, allowing us to capture significant geographic and environmental heterogeneity. Our study makes two primary contributions to the literature. First, we conduct a county-level analysis that decomposes dairy TFP into separate elements, explicitly distinguishing the direct effect of heat stress, measured by THI and indirect climatic impacts on feed captured by GDD and precipitation. Second, we report regional heterogeneity in these components across major U.S. dairy regions, insinuating the importance of localized adaptation policies.

The last four decades have seen extensive on-farm adaptation of new technologies and management practices in the U.S. dairy sector. On the biological side, technological progress has involved sustained genetic improvement in Holstein dairy cattle and, more recently, targeted breeding for greater resilience and thermotolerance [4,16,55,56,57], alongside the use of selected biotechnologies such as recombinant bovine somatotropin (rBST) [58]. On the physical and management side, adaptation has relied on investments in cooling and environmental control [7,8,52,59], and on advances in barn design and cow comfort that support both productivity and animal welfare [60,61,62]. Another dimension has been the diffusion of on-farm microclimate and herd-level monitoring tools that enable more precise heat-stress management [63,64]. Data limitations do not allow us to explicitly model individual technologies; their combined effects are reflected in the technological progress component and the climatic indices. Our analysis indicates that how individual biological, physical, and informational innovations interact with climatic stressors at the farm level is an important area for future work.

The remainder of this paper is organized as follows: Section 2 details the dataset and empirical methodology used. Section 3 presents the results and discussion, and Section 4 offers a summary of our findings and concluding remarks. Given the vital role of productivity in shaping agricultural policy, our findings provide insights for policymakers and stakeholders in the United States and beyond.

## 2. Materials and Methods

### 2.1. Materials

This study utilized county-level data from the USDA Census of Agriculture, published every five years by NASS. The dataset spans the years 1978 through 2022 and includes comprehensive county-level information for all U.S. counties with at least USD 1000 in annual farm sales [65]. These data provide detailed information and broad geographical coverage offering a valuable alternative to farm-level analysis that relies on data that is often difficult to access [58,66,67,68,69,70].

**Figure 1:**
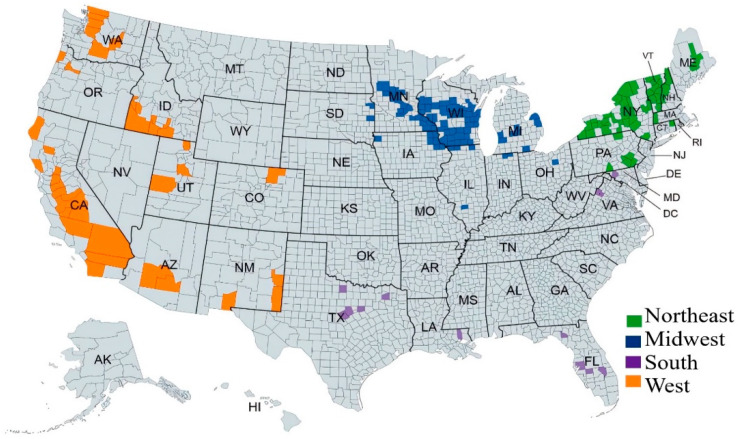
Dairy counties and regions.

The county selection process followed the methodology of Njuki and Bravo-Ureta [69], which prioritizes the top 100 counties in terms of dairy cow inventories as reported in the “State and County Rankings” volume of each agricultural census. Following their approach, we also included counties where dairy sales constituted at least 30% of total agricultural sales for each census year included in the analysis. This procedure yielded 179 dairy counties (Figure 1), and after excluding observations with missing data, our final unbalanced panel comprised 1783 county-level observations. Those counties are distributed across four regions: Midwest, Northeast, South, and West, as shown in Figure 1, following the definitions used by the USDA NASS to collect agricultural census data [65].

Our analysis employed carefully defined output and input variables to ensure robust estimation. We consistently measured *milk equivalent*, derived by dividing the total value of dairy product sales by the relevant state-based unit milk price for the period. Counties in our sample produced an average of 264,424 tons of milk equivalent annually (Table 1).

We disaggregated conventional inputs as follows: *dairy cows* represent the average number of dairy cows per county, averaging 29,856 head. However, this national average mask significant variation across regions and census periods, as detailed in Table A1 of Appendix A. Notably, the West leads in dairy cow population, which has seen substantial growth over the past 45 years, while the South consistently exhibits the lowest numbers. *Farm labor* accounts for both hired and contract labor hours, averaging 1.1 million hours annually per county. *Farm expenses* encompass outlays for livestock inputs, fertilizers, and chemicals utilized explicitly in dairy farming, averaging USD 14.6 million per county (Table 1). We measured *concentrate feed* (16% protein) at an average of 120.8 tons per county. Finally, we represented *machinery* by the value of equipment associated with milk production, averaging USD 67.4 million per county per year (Table 1). We deflated all monetary figures, including farm expenses and machinery values, to constant 2022 dollars using the Price Indices and Implicit Quantities of Farm Output and Inputs dataset published by the USDA Economic Research Service (ERS) [71].

We utilized county-level weather data for the period 1978–2022 from the Visual Crossing Weather Database (VCWD) [72]. From this database, which provides consistent information for two-kilometer grid cells, we compiled daily minimum, maximum, and average temperatures, relative humidity, and monthly precipitation. We employed three alternative variables to represent climatic effects: growing degree days (GDD); annual precipitation; and the Temperature-Humidity Index (THI) load (Table 1).

GDD measures cumulative heat exposure for a crop within a specific temperature range, calculated by summing daily heat over the growing season (1 April to 30 September). Specifically, *GDDi* = $\sum_{d=1}^{D}\{\min\left[\left(\frac{T_{max}+T_{min}}{2}\right),T_{ub}\right]$ − *T_lb_*}*_di_*, where *T_max_* and *T_min_* are daily maximum and minimum temperatures (°C), T_ub_ (30 °C) and *T_lb_* (10 °C) are the upper and lower temperature bounds, and *D* is the number of days in the growing season [70]. Maize was selected as the reference crop for GDD calculations because corn silage is the primary forage source in U.S. dairy rations and is highly sensitive to thermal conditions [58]. The inclusion of GDD is critical because it directly reflects the thermal requirements for feed crops, is well-established in the scientific literature, and is essential for capturing the environmental heterogeneity that drives regional differences in dairy TFP [51,73]. Consequently, it provides a pertinent measure of the interplay between climatic effects and productivity. The annual county-level GDD average is 1578. We also included annual precipitation in our model as another key climatic variable. Sufficient precipitation ensures a reliable water supply for both direct cow consumption and the cultivation of feed crops [54,58]. Conversely, drought conditions severely constrain milk production by reducing forage yields, exacerbating heat stress, and increasing operational costs [58]. Therefore, this variable serves as a key indicator for feed availability and environmental stress, which are fundamental drivers of dairy productivity [74,75]. The average annual precipitation is 717 mm (Table 1).

The THI load is the cumulative sum of THI when it exceeds 72 annually and can be defined as Equation (1):

(1) $$THI=(1.8*T_{dbit}+32)-(0.55-0.0055*{RH}_{it})*(1.8*T_{dbit}-26)$$

where *T_dbit_* is the dry-bulb temperature (°C) and *RH_it_* is the relative humidity (%). The THI load is defined as the cumulative sum of daily THI values above a threshold of 72. Formally,

(2) $${THI\;load}_{i}=\sum_{d=1}^{D}\{{THI}_{max}-{THI}_{72}{\}}_{di}$$

where *THI_max_* is the maximum daily THI on day *d*, and *THI*_72_ is a threshold value of 72 [16]. Because the physiological impact of heat stress is cumulative, simple annual averages can be misleading by masking the effect of intense, prolonged heat waves [22,55]. Therefore, following Key and Sneeringer [30], we employed the THI load, which quantifies both the duration and magnitude of heat exposure above a critical threshold, offering a more biologically relevant measure of the total heat burden experienced by dairy herds. The annual average THI load is 101 (Table 1). Table A2 of Appendix A presents the mean THI load values by year and region. Although the South exhibits a higher average THI load, the West has experienced a more pronounced increase, particularly over the last decade.

The empirical framework accounts for unobserved heterogeneity by grouping counties into the four regions and incorporating regional fixed effects (Table 1). To account for temporal shifts arising from technological progress, we also included time fixed effects for each census year (Table 1) [41,49].

### 2.2. Methods

#### 2.2.1. Cobb–Douglas Stochastic Production Frontier

This study employs the stochastic production-frontier (SPF) approach to examine how different variables influence milk output and productivity across U.S. dairy counties. Since Aigner et al. [76] and Meeusen and van den Broeck [77] independently introduced the SPF framework, it has become a staple in agricultural economics, including dairy research [78,79,80,81,82]. Within this SPF framework, we adopt a Cobb–Douglas (CD) functional form rather than the more flexible translog (TL) specification. In principle, the TL can capture richer interaction effects among inputs, but the CD specification, under suitable parameter restrictions, guarantees global non-negativity and monotonicity, and its coefficients can be interpreted directly as output elasticities, which facilitates economic interpretation [38,53]. Moreover, TFP measures derived from a CD function satisfy the transitivity property, an essential requirement from index-number theory for consistent productivity comparisons across regions and over time [38,53]. So, we adopt the C-D considering these conceptual issues and its good performance in related stochastic frontier applications.

We make a distinction between production technology and the characteristics of the production environment. O’Donnell [53] defines the “production technology “as a way, method, or system that transforms inputs into outputs” (p. 2). In contrast, environmental characteristics are variables that are outside the firm’s control but physically involved in the production process (e.g., weather, geographic location, topography). Following O’Donnell [53], the production technology can be expressed as (Equation (3)):

(3) $$P^{t}\left(z\right)=\left\{\left(x,q\right):x\;can\;produce\;q\;in\;period\;t\;in\;environment\;z\right\}$$

To address the inflexibilities of the traditional CD model, which imposes fixed output elasticities across all observations, we apply a Random Parameters Cobb–Douglas specification [41]. We then employ the estimates of the random parameters (RP) to examine the effects of climatic variables on the productivity of dairy counties. Unlike traditional SPF models, the random parameters model also accounts for technological and environmental heterogeneity [83,84]. With this approach, we measure the heterogeneous responses of each dairy county to both conventional inputs and changes in climatic effects [41]. Following Julien et al. [85] and Bravo-Ureta et al. [42], a CD representation of the RP model can be expressed as:

(4) $$\ln\;y_{it}=\alpha_{i}+\sum_{n=1}^{5}\beta_{n}\ln X_{nit}+\sum_{j=1}^{3}\rho_{jit}{lnZ}_{jit}+\sum_{t=1978}^{2022}\gamma_{t}D_{t}\;+\;v_{it}-u_{it}$$

where *y_it_* is milk output for the *ith* county in year t, $\alpha_{i}$ is a region level fixed effect, and *X_nit_* are conventional inputs, namely number of cows, feed, labor, machinery-equipment, and farm expenses. *Z_jit_* are climatic effects, namely GDD, annual precipitation, and THI load. $D_{t}$ represents time dummy variables corresponding to the specific census years: 1978, 1982, 1987, 1992, 1997, 2002, 2007, 2012, 2017, and 2022, which are included to capture technological change. The Greek characters *β*, *ρ*, and *γ* are parameters to be estimated. The random parameter *α_i_ ≡ α*(z_i_^*^) in the model shows the effect of unobserved heterogeneity that does not change over time, while *β_it_ ≡ β*($z_{it}^{*}$) and *ρ*_it_ ≡ *ρ* ($z_{it}^{*}$) capture time-varying heterogeneity, allowing for county-specific responses to inputs and climatic effects. Their respective distributions are ρ*_jit_ ∼ N* (ρ*_j_*, $\sigma_{\rho j}^{2}$), $\beta_{nit}$ ∼ *N* (*β_n_*, $\sigma_{\beta n}^{2}$) [42]. Finally, v_it_ is the statistical noise with a normal distribution, *v_it_* ∼ *N* (0, *σ*^2^_*v*_), and *u_it_* is an inefficiency term with a half-normal distribution, *u_it_* ∼ *N* + (0, *σ*^2^_*u*_) [49,76]. We calculate the technical efficiency component for each observation given by *TE_it_ = exp* (−*û_it_*), following Jondrow et al. [86].

#### 2.2.2. Total Factor Productivity Index (TFPI) and Components

Productivity is equal to output quantity relative to the total amount of inputs [49]. A TFPI is given by the ratio of the productivity of firm *i* in period *t* relative to firm *k* in period *s* and can be represented as:

(5) $$TFPI=\frac{\left[Q(q_{it})/X(x_{it})\right]}{\left[Q(q_{ks})/X(x_{ks})\right]}$$

In this analysis, we use the general index developed by O’Donnell [53] to calculate sources of productivity growth in the U.S. dairy counties. Following Bravo-Ureta et al. [42], the complete representation of TFP under the random parameter model is given by:

(6) $${TFPI}_{ksit}=\left[\frac{{\exp\gamma }_{t}}{{\exp\gamma }_{s}}\right]\left[\prod_{n=1}^{N}\left(\frac{x_{nit}^{\beta_{nit-bn}}}{x_{nks}^{\beta nks-bn}}\right)\right]\left[\prod_{j=1}^{J}{\left(\frac{Z_{jit}}{Z_{jks}}\right)}^{\rho_{j}}\left(\frac{exp\left(\alpha_{i}\right)}{exp\left(\alpha_{k}\right)}\right)\right]\left[\frac{exp\left(u_{it}\right)}{exp\left(u_{ks}\right)}\right]\left[\frac{exp\left(v_{it}\right)}{exp\left(v_{ks}\right)}\right]$$

where the first component on the right-hand side of the equation is the output-oriented technological progress index (TP), which captures the change in productivity associated with shifts in the production frontier due to the discovery and application of new technologies over time. The second component is the output-oriented scale efficiency index (OSEI), reflecting changes due to economies of scale, where $b_{n}=\hat{\beta_{n}}/\sum_{k=1}^{N}\hat{\beta_{k}}$ is an estimator of the mean of the distribution of *β_nit_*. The third component is the environmental index (EI), which accounts for weather variables and time-invariant region-specific heterogeneity. We express this index as two components: EI-THI, which captures changes in growth due to heat stress (THI > 72); and EI-FEED, indicating changes in productivity that come from GDD and precipitation. The fourth component is the output-oriented technical efficiency index (OTEI), which measures management performance and reflects movements toward the production frontier. The last component is the statistical noise index (SNI), which identifies changes in productivity that are not accounted for by the model.

To measure the change in TFP over time for all components, we take one county, Maricopa, AZ, in 1978, as the reference point. We selected this county systematically, as it is the first observation when our sample is sorted by the Federal Information Processing System code, thus ensuring an objective point of reference. It is important to reiterate that under O’Donnell’s [53] index-number framework, transitivity ensures that the choice of a reference observation does not affect the relative rates of change in TFP across counties or over time. The rate of change in TFP is revealed by comparing the other years and all remaining counties with the reference county in 1978.

## 3. Results and Discussion

Before presenting the detailed analysis of TFP measures and their components, this section outlines the model specifications we compared, which support selecting the preferred Random Parameters Stochastic Production Frontier (RP-SPF) model through hypothesis testing.

### 3.1. Definition of Models and Hypothesis Tests

Having estimated various models, we report the results for the two most relevant specifications.

Model 1: Pooled-SPF that includes the conventional inputs, three climatic indicators, regional fixed effects, and time dummy variables. Model 1 assumes that all dairy counties share the same production technology.

Model 2: RP-SPF that incorporates the conventional inputs, climatic effects, regional fixed effects, and time dummy variables. The parameters for cows, labor, farm expenses, feed, and machinery inputs, and GDD, precipitation, and THI load are assumed to be random, while the parameters of the regional fixed effects and time dummies are treated as non-random (Equation (4)). Model 2 extends Model 1 by incorporating random parameters, which account for unobserved county-level heterogeneity.

To test the hypothesis that the estimated coefficients of the conventional inputs and climatic effects are random, we compared Model 1 (Pooled-SPF) and Model 2 (RP-SPF). Model 1 is nested within Model 2, serving as the restricted (R) specification, while Model 2 is the unrestricted (UR) specification. Because the R model is nested in the UR model [87], we use a likelihood ratio (LR) test (Table 2). The LR statistic is calculated as LR = −2[lnL_R_ − lnL_UR_], which follows a chi-square (χ^2^) distribution with J degrees of freedom (the number of restrictions) [87]. We compare this statistic with the critical values from Kodde and Palm [88] and reject the null hypothesis (H_0_) of the restricted model if the LR value exceeds the critical value [89]. The likelihood values for Models 1 and 2 are 1826 and 2055, respectively (Table 3), with 8 degrees of freedom corresponding to the five conventional inputs and three climatic variables for which random parameters are estimated. The LR statistic is 458 (*p* < 0.001), which exceeds the 5% critical value; therefore, we reject H_0_ and select the unrestricted Model 2 for the analysis.

We clustered the standard errors at the county level and estimated all RP models using simulated maximum likelihood methods with 100 Halton draws in NLogit version 6 and then generated the means of the random parameter estimates for the analysis by averaging the individual parameters across all counties and time periods. Table 3 reports the estimated means of the random parameters, which incorporate the conventional inputs and the climatic effects. The table also provides model fit statistics such as the Akaike Information Criterion (AIC) and Log-Likelihood values as well as the parameters for the error components.

### 3.2. Parameter Estimates of the RP Models

In our preferred specification (Model 2), the parameters of the five conventional inputs are positive, as expected, and are significant at the 1% level. We used the Wald test for constant returns to scale (i.e., Σ*β_m_* = 1) [87,88]. This hypothesis is rejected (*χ*^2^ = 49.90, *p* < 0.001), indicating mildly increasing returns to scale for Model 2 (Table 2). The parameters for all climatic variables are also significant. The time dummies are significant at the 1% level, as are those for the regional fixed effects in Models 1 and 2 (Table 3).

We also estimated alternative model specifications. One alternative model replaced regional fixed effects with county-specific latitude and longitude coordinates, which performed poorly and was discarded.

By contrast, Model 2 incorporates regional fixed effects instead of continuous coordinates [90,91]. This approach helps address collinearity and allows the model to account for time-invariant, region-specific factors that the geographical coordinates may not capture. We therefore treat Model 2 as the most robust specification and focus on it below.

The output elasticities from Model 2 confirm that the number of cows is the most important conventional input (0.720), consistent with the literature [49,92,93]. A 1% increase in herd size raises milk output by 0.72%. The elasticities for labor (0.105), farm expenses (0.066), feed (0.056), and machinery (0.084) are also positive. The elasticity of machinery exceeds that of concentrate feed, reflecting the capital-intensive nature of modern U.S. dairy systems [58]. Additional investments in machinery are therefore associated with stronger output responses than comparable increases in feed use.

The parameters for the climatic variables are also statistically significant. Favorable conditions for feed cultivation, such as a 1% increase in GDD and precipitation, are associated with output increases of 0.05% and 0.01%, respectively, which aligns with existing studies [49,58]. These factors indirectly affect milk output by influencing feed quantity and quality [58,94]. The estimated elasticity of output with respect to the THI load is −0.011, which appears small at the mean. Given that the average THI load is about 101 (SD = 183; Table 1), a one-standard-deviation increase in THI load is associated with an output reduction of about 2.0% (183 × 0.011 ≈ 2.0%). This result is consistent with earlier research showing that higher THI levels reduce milk output [30,47,55].

Finally, both regional and time-fixed effects (the South region and the 1978 census year as the excluded categories) are statistically significant. The likelihood ratio test confirms the importance of regional fixed effects (*p* = 0.000), with results showing that the West has the most favorable production environment (relative to the South baseline). This pattern is consistent with the long-run geographic transformation of the U.S. dairy sector, whereby cow inventories have shifted from traditional production regions in the Midwest and Northeast towards Western states [67]. Western dairy production has moved toward a highly industrialized model, characterized by larger herd sizes and substantial investment in large-scale, technologically advanced facilities [67].

Similarly, the positive time-fixed effects for each census period after 1978 point to robust technological progress with estimated productivity growth peaking at 74% in the 2017–2022 period relative to the 1978 baseline.

Regarding the model’s error structure, the parameter lambda (*σ_u_/σ_v_*) is equal to 2.99 (Table 3), indicating that the variance of the one-sided error component is slightly larger than that of the two-sided component [95]. This highlights the statistical significance of technical inefficiency on milk output changes [38]. The annual average TE score estimated from Model 2 is 92.4% (Table 4). This score is consistent with the high TE scores of previous dairy studies reported in the meta-analysis by Bravo-Ureta et al. [96] and Moreira Lopez and Bravo-Ureta [80].

The analysis indicates that U.S. dairy counties are, on average, highly efficient, achieving 92.4% of their maximum potential output given their resources and technology. This high average is driven by a large concentration of well-managed operations clustered at the top end of the performance scale, suggesting best practices in resource use [97,98].

**Figure 2:**
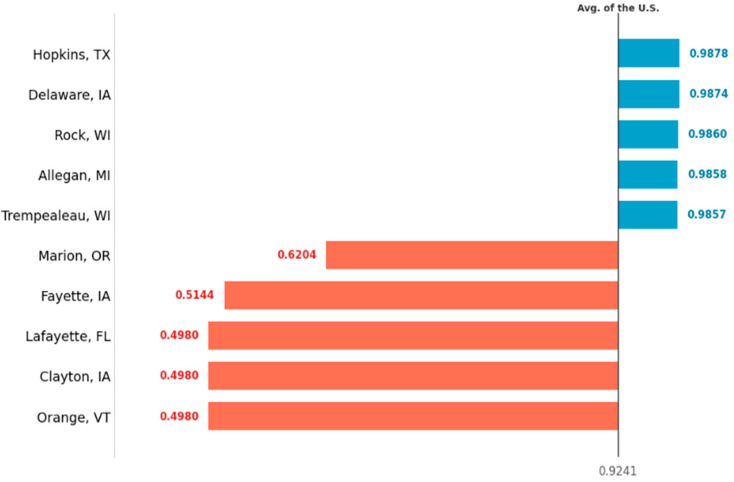
Comparison of Technical Efficiency (TE) scores for the top 5 (blue) and bottom 5 (red) U.S. dairy counties. The vertical line indicates the U.S. average TE score (0.9241).

However, as illustrated in Figure 2, the high average TE masks a critical efficiency gap. While the top-performing counties achieve high TE scores (>0.98), Figure 2 identifies a “tail” of underperforming operations, in several counties (e.g., Lafayette, FL; Orange, VT) operating at TE scores below 0.63, far below the national average. From an industry perspective, this “tail” of underperforming counties represents an untapped potential for productivity and output growth.

### 3.3. Total Factor Productivity (TFP) Measures and Its Components

The analysis of the TFP index (TFPI) and its components (Table 5) reveals a strong increasing trend in U.S. dairy productivity after 1978, peaking in 2022. Following O’Donnell [53], the TFPI is calculated as TFPI = TP × OSEI × EI-FEED × EI-THI × OTEI × SNI. For example, the U.S. TFPI for the base year of 1978 was calculated using the 1978 components from Table 5 as follows: TFPI = 1.000 × 0.966 × 0.931 × 1.018 × 0.999 × 1.037 = 0.949). The highest five-year growth rate of 15.5% was achieved between 2007 and 2012. This growth was primarily driven by TP, which displayed a consistent upward trend and reached its highest level (2.99%) in the final period. The largest surge in TP occurred between 1978 and 1982 at 60.1%.

This progress reflects an evolution of productivity–enhancing innovations promoting adaptation–oriented investments [56,58]. Earlier decades were characterized mainly by gains in genetics and mechanization [4,16], whereas from the 1980s onward the rapid expansion of cooling infrastructure, improved barn environments, and microclimate monitoring systems became critical for sustaining TFPG under rising heat loads [7,8,62,63,99]. The pivotal role of TP is underscored in the 2002–2007 period, where a temporary decline in TFPG was directly attributable to a 4.6% decrease in TP. The decline in the 2002–2007 period coincides with rapid structural change in the U.S. dairy sector, as production shifted toward larger capital-intensive farms in the West [100]. While these operations ultimately achieve lower unit costs, the transition process entails short-run adjustment—such as farm exits and capital reallocation—that can temporarily depress productivity. Our results are therefore consistent with an evolving structural reorganization rather than a permanent reversal of technological progress.

In contrast to the steady gains from technological progress, average OSEI fluctuated before peaking in 1982 and then exhibited a slight decreasing trend. Similarly, the average OTEI showed a slight decline over the 45 years, suggesting a modest decrease in managerial performance, with the largest drop (2.7%) occurring between 2017 and 2022.

The environmental indices reveal mixed effects on productivity. The average EI-FEED showed an overall increasing trend despite modest fluctuations, including a 0.8% decline in the 1987–1992 subperiod that corresponded with declines in GDD averages (Table A3 in Appendix A). On the other hand, EI-THI declines overall but exhibits sharp fluctuations over time (e.g., −1% in 1992–1997). The most pronounced decline occurs between 1992 and 2002. These swings—for instance, the drop between 1992 and 1997—are consistent with observed variation in THI load across regions (Table A2 in Appendix A), reflecting the influence of heat waves and interannual climate variability on the environmental component of TFP. Based on this overview, we now turn to the year-to-year dynamics of TFPI and its components and examine the main drivers of these changes.

Table 6 presents the average annual TFPG for the U.S. and its regions over the 45-year period. The average annual TFPG for the U.S. was 1.59%, driven primarily by TP at a 2.52% per year (Table 6). These results are consistent with other dairy studies. For example, Njuki et al. [49] found TFPG of 2.16% in Wisconsin, while a nationwide study by Njuki [58] identified annual TFPG of 2.51%.

While technology was the primary driver of TFPG, other components made smaller contributions. Climatic effects played a dual role; favorable feed conditions (EI-FEED) provided a modest annual increase of 0.02% to TFPG. This positive effect is directionally consistent with Njuki [58], who found a more pronounced contribution of 2.2% from feed-related climatic factors. The difference in magnitude likely stems from our use of county-level census data over a longer timeframe versus Njuki’s farm-level survey over a shorter period, underscoring the value of examining climatic impacts at different aggregation scales to capture both granular farm dynamics and broader long-run regional trends.

In contrast, the impact of heat stress (EI-THI) on the rate of productivity growth was minimal, reducing TFPG by 0.01% annually. This low magnitude is consistent with the hypothesis that widespread on-farm adaptation strategies have mitigated part of the negative effects of heat stress [101,102]. However, other studies show that these strategies have not fully neutralized the impact of heat, which still results in substantial economic losses for the U.S. dairy industry, with annual estimates ranging from USD 897 million to USD 1.5 billion [3,57,62,103]. Meanwhile, efficiency change has been slightly negative. OSEI decreased at an average rate of 0.01% per year, consistent with other recent studies [49,58].

The slightly negative trend in OTEI (−0.091%) suggests a modest weakening in average managerial performance rather than a sharp decline. Although our framework cannot identify specific mechanisms, recent evidence on U.S. dairy consolidation [104] points to plausible sector-level influences, including increased reliance on hired labor, uneven adoption of productivity-enhancing technologies, and the continued operation of high-cost farms. These structural features may contribute to the negative OTEI observed in our analysis. Finally, the SNI component averaged −0.83% (Table 6).

A detailed breakdown by region (Table 6) and by state (Table A4 in Appendix A) reveals significant performance disparities. The Midwest (1.74%) was the fastest-growing region, led by South Dakota (1.93%), in contrast with Iowa (1.46%), the slowest-growing state. This pattern is consistent with documented outreach efforts in the Northern Great Plains, where producers have been actively managed heat stress by optimizing environmental control systems to enhance cow comfort and productivity [105]. These initiatives focus on fan maintenance, airflow pattern detection, and animal monitoring, reflecting a proactive approach to mitigating heat stress impacts [105].

The Northeast (1.53%) shows wide variation, from Massachusetts (1.75%) to Vermont (1.20%), highlighting that farm-level strategies can be more decisive than broad climatic trends [106]. The South (1.33%) was the slowest-growing region [107], with Texas (1.80%) and Florida (0.58%), the nation’s slowest-growing state. The West (1.42%) showed the benefits of OSEI and adaptation, with New Mexico (1.88%) and Colorado (1.86%) achieving high growth despite climatic pressures, supported by sophisticated management practices [108] and advanced technologies [109].

**Figure A1:**
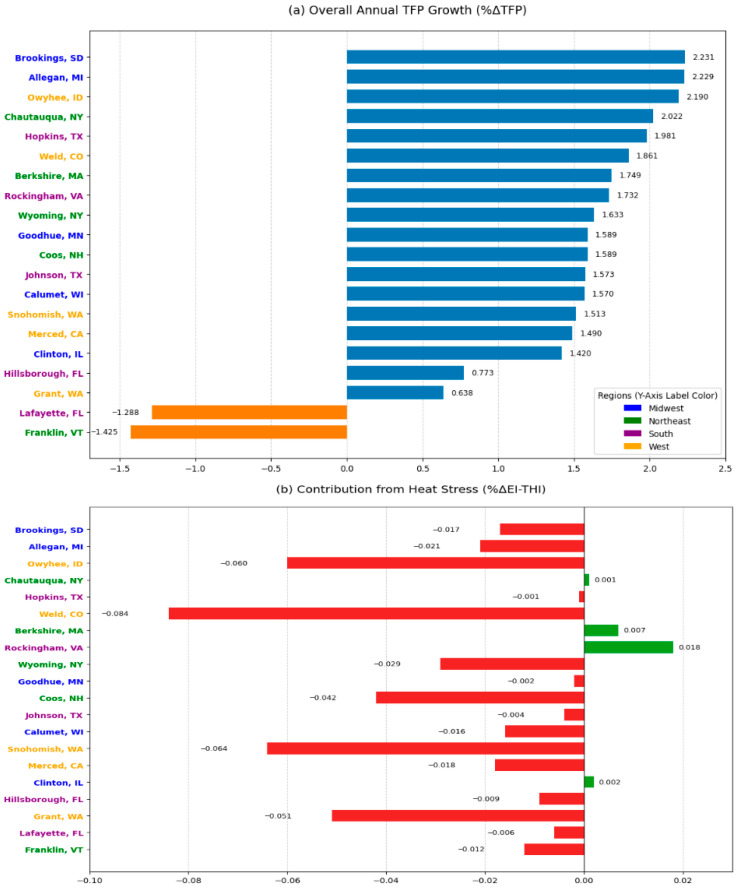
Comparison of TFP growth and heat stress contributions in selected U.S. dairy counties (1978–2022): (**a**) overall annual TFP growth (%ΔTFP), (**b**) contribution from heat stress (%ΔEI-THI).

Figure A1 of Appendix A demonstrates the counties with the highest and lowest TFPG along with a sample of selected counties. The county-level analysis highlights a wide range of outcomes, underscoring that local adaptation and resilience can play a significant role. A noteworthy pattern is that some of the fastest-growing counties also face significant climatic constraints. For example, Brookings, SD (Midwest), and Owyhee, ID (West), achieved some of the nation’s highest annual TFPG rates, at 2.23% and 2.19%, respectively (Figure A1a in Appendix A). This occurred even as they experienced notable TFP reductions from heat stress (−0.02% for Brookings and −0.06% for Owyhee) (Figure A1b in Appendix A), suggesting that good management and technology adoption can more than compensate for adverse environmental pressures. This contrasts with counties like Lafayette, FL (South), and Franklin, VT (Northeast), which saw negative overall TFPG, at −1.28% and −0.42%, respectively (Figure A1a in Appendix A). The most severe climatic challenge was observed in Weld, CO (West), where heat stress reduced annual TFPG by 0.08% (Figure A1b in Appendix A). However, the county still achieved a strong overall growth rate of 1.86% (Figure A1a in Appendix A), further demonstrating the decisive role of adaptation in mitigating climatic impacts.

Our findings show that the U.S. dairy sector has maintained positive TFP growth despite rising heat loads. The combination of robust technological progress (2.52% annually), high average technical efficiency (92.4%), and the relatively small constraints from climatic variables—particularly heat stress—suggests that farmers have implemented effective adaptation strategies [47]. Studies in the Southeast and Wisconsin demonstrate that direct mitigation measures, such as cooling systems, significantly reduce production losses and generate benefits that exceed their costs [47,110].

Our results also align with international evidence from other regions facing similar climatic pressures, strengthening the external validity of our conclusions. Research from Southern Europe, Australia, and Latin America consistently shows that, while heat stress and adverse weather slow growth, underlying productivity trends remain positive in well-adapted systems [19,28,37,44,111,112].

This resilience requires ongoing investment [59,110,113]. The limited overall effect of climate on TFP likely reflects both these direct adaptations and broader, often costly, managerial changes, including precision data systems and improved feeding strategies [59,108,109,110,113,114]. This pattern underscores the importance of management in balancing avoided losses with additional operating costs. Future research should move beyond aggregate indices to quantify the farm-level economic returns of specific adaptation strategies and identify which combinations of technologies and practices offer the greatest resilience per unit cost.

## 4. Conclusions

This study provides a comprehensive analysis of total factor productivity growth (TFPG) across 179 U.S. dairy counties from 1978 to 2022, with a specific focus on climatic factors. We utilize publicly available county-level panel data from USDA NASS, which offer a distinct advantage over comparable farm-level panel data with restricted access. We employ the RP-SPF framework to decompose TFPG. The analysis yielded two principal findings. First, technological progress has been the primary driver of productivity growth, averaging 2.52% annually. Second, while climatic factors pose challenges, their net impact on TFPG has been low. Our model disaggregated this into a modest positive contribution from feed-related conditions (EI-FEED) and, most notably, a low national-level drag from direct heat stress (EI-THI).

Our framework explains changes in total factor productivity; however, it does not isolate the specific contribution of individual strategies—such as cooling systems, precision feeding, or breeding for thermotolerance—from overall technological progress. Additionally, structural changes within the sector and potential implications for animal welfare are only indirectly reflected in our aggregate indices. Consequently, our conclusions regarding adaptation rely on indirect evidence.

In conclusion, this research indicates that the U.S. dairy sector has maintained a positive productivity trend despite increasing climatic pressures. The sector has successfully leveraged technological change and adaptive management to buffer the adverse effects of a warming climate. The long-term sustainability of these gains will depend on two key factors: first, future research using farm-level data to directly quantify the economic returns of specific adaptation schemes, and second, continued innovation and policies that support the diffusion of the most effective strategies across diverse U.S. dairy systems.

## Figures and Tables

**Table 1:** Variable Definitions and Descriptive Statistics.

Variables	Abbr.	Description	Mean	SD
Output	(*Y_t_*)	Milk equivalent (tons)	264,424	408,527
*Conventional inputs*				
Dairy cows	(*X*_1_*_it_*)	Number of dairy cows (head)	29,856	38,567
Farm labor	(*X*_2_*_it_*)	Total labor hours (000 h)	1,108,813	2,067,296
Farm expenses	(*X*_3_*_it_*)	Total value of livestock inputs, chemicals, and fertilizer expenses (000 USD)	14,608	18,477
Concentrated feed	(*X*_4_*_it_*)	Quantity of 16% protein concentrate feed (tons)	121	263
Machinery	(*X*_5_*_it_*)	Machinery-equipment value (000 USD)	67,356	52,131
*Climatic variables*				
GDD	(*Z*_1_*_it_*)	Number of GDD (days)	1578	420
Precipitation	(*Z*_2_*_it_*)	Annual precipitation (mm)	717	397
THI load	(*Z*_3_*_it_*)	The cumulative sum of THI when it exceeds 72 during the relevant year	101	183
*Regional Fixed Effects*				
Northeast	(*α*_1_)	Regional dummy = 1, if the county is in the Northeast region	0.268	0.443
Midwest	(*α*_2_)	Regional dummy = 1, if the county is in the Midwest region	0.442	0.497
South ^(a)^	(*α*_3_)	Regional dummy = 1, if the county is in the South region	0	0
West	(*α*_4_)	Regional dummy = 1, if the county is in the West region	0.223	0.416
*Time Fixed Effects*				
1978 ^(b)^	(*D_t_*_1_)	Time dummy = 1, if the year is 1978	0	0
1982	(*D_t_*_2_)	Time dummy = 1, if the year is 1982	0.100	0.300
1987	(*D_t_*_3_)	Time dummy = 1, if the year is 1987	0.100	0.301
1992	(*D_t_*_4_)	Time dummy = 1, if the year is 1992	0.100	0.301
1997	(*D_t_*_5_)	Time dummy = 1, if the year is 1997	0.100	0.301
2002	(*D_t_*_6_)	Time dummy = 1, if the year is 2002	0.100	0.301
2007	(*D_t_*_7_)	Time dummy = 1, if the year is 2007	0.100	0.301
2012	(*D_t_*_8_)	Time dummy = 1, if the year is 2012	0.100	0.300
2017	(*D_t_*_9_)	Time dummy = 1, if the year is 2017	0.100	0.301
2022	(*D_t_*_10_)	Time dummy = 1, if the year is 2022	0.099	0.298

Note: Statistics are based on an unbalanced panel of 179 U.S. dairy counties over ten USDA census years (1978–2022), totaling 1783 observations. All monetary values are expressed in constant 2022 dollars. ^(a)^ and ^(b)^ show excluded region and year dummy variables, respectively. Source: Authors’ calculations based on data from the USDA Census of Agriculture and VCWD.

**Table 2:** Hypothesis Tests.

Model Selection	Null Hypothesis	Test Statistic	*p*-Value	Decision
*Log-likelihood Ratio Test*				
Model 2 vs. Model 1	Estimated parameter of conventional inputs and climatic variables are equal to zero ( $\hat{\beta_{1}}=\hat{\beta_{2}}=\hat{\beta_{3}}=\hat{\beta_{4}}=\hat{\beta_{5}}=0$) ($\hat{\rho_{1}}$ = $\hat{\rho_{2}}$ = $\hat{\rho_{3}}$ = 0).	458.12	<0.001	Reject null hypothesis
*Wald Test*				
Return to scale (for Model 2)	Constant returns to scale prevail if $\sum_{m}\hat{\beta_{m}}=1$.	49.90	<0.001	Reject null hypothesis

**Table 3:** Coefficient Estimates of Pooled and Random Parameter SPF models.

Variables	Model 1 (Pooled SPF)	Model 2 (RP-SPF)
Constant	1.063 ***	0.927 ***
	[0.268]	[0.139]
Dairy cows	0.762 ***	0.720 ***
	[0.017]	[0.007]
Labor	0.092 ***	0.105 ***
	[0.009]	[0.004]
Farm expenses	0.064 ***	0.066 ***
	[0.008]	[0.003]
Feed	0.057 ***	0.056 ***
	[0.008]	[0.003]
Machinery	0.052 ***	0.084 ***
	[0.012]	[0.005]
GDD	0.054 ***	0.054 ***
	[0.027]	[0.019]
Precipitation	0.010 ***	0.010 ***
	[0.027]	[0.004]
THI load	−0.011 ***	−0.011 ***
	[0.026]	[0.003]
Time Fixed Effects	Yes	Yes
Regional Fixed Effects	Yes	Yes
Number of Observations	1783	1783
Number of counties	179	179
Sigma(u)	0.118	0.11
Sigma(v)	0.054	0.037
Lambda	2.178 ***	2.994 ***
	[0.159]	[0.217]
LL Function	1825.96	2055.02
AIC	−3605.9	−4046
Avg. Technical Efficiency	0.917	0.924
Returns to Scale	1.031	1.031

Note: Robust standard errors are in brackets; *** denotes significance level at 1%.

**Table 4:** Technical efficiency scores: Descriptive Statistics.

	Mean	Std. Deviation	Min	Max	Skewness	Kurtosis
TE scores	0.924	0.047	0.498	0.988	−2.812	20.174

**Table 5:** Quinquennial Decomposition of the U.S. Total Factor Productivity Index.

USDA Census Year	TFPI	TP	OSEI	EI-FEED	EI-THI	OTEI	SNI
1978	0.949 (-)	1.000 (-)	0.966 (-)	0.931 (-)	1.018 (-)	0.999 (-)	1.037 (-)
1982	1.034 (9.0)	1.601 (60.1)	0.968 (0.2)	0.936 (0.6)	1.020 (0.2)	1.000 (0.0)	0.699 (−9.4)
1987	1.179 (14.1)	1.821 (13.7)	0.967 (−0.2)	0.941 (0.5)	1.014 (−0.6)	0.996 (−0.4)	0.706 (0.2)
1992	1.315 (11.5)	2.007 (10.2)	0.965 (−0.2)	0.933 (−0.8)	1.032 (1.8)	0.996 (0.0)	0.708 (0.1)
1997	1.447 (10.0)	2.227 (10.9)	0.965 (−0.1)	0.935 (0.1)	1.022 (−1.0)	0.996 (0.1)	0.708 (0.0)
2002	1.616 (11.7)	2.513 (12.8)	0.965 (0.0)	0.939 (0.5)	1.012 (−1.0)	0.993 (−0.3)	0.706 (−0.1)
2007	1.528 (−5.5)	2.396 (−4.6)	0.966 (0.1)	0.940 (0.1)	1.018 (0.6)	0.982 (−1.1)	0.702 (−0.1)
2012	1.765 (15.5)	2.752 (14.8)	0.965 (−0.1)	0.941 (0.1)	1.015 (−0.4)	0.986 (0.3)	0.706 (0.1)
2017	1.874 (6.2)	2.907 (5.6)	0.965 (0.0)	0.940 (−0.1)	1.024 (0.9)	0.986 (0.1)	0.704 (−0.1)
2022	1.897 (1.2)	2.994 (3.0)	0.963 (−0.2)	0.941 (0.2)	1.015 (−0.9)	0.959 (−2.7)	0.718 (0.4)
Cumulative % Change (1978–2022)	(99.9)	(199.4)	(−0.3)	(1.1)	(−0.3)	(−4.0)	(−30.8)

Note: The five-year percentage change for the TFPI and its components is calculated using this formula: ((TFPI_t_ − TFPI_t−5_)/TFPI_t−5_ × 100)) [93]. For example, the 15.5% change in TFPI between 2007 and 2012 is calculated as (1.765 − 1.528)/1.528) × 100; Those changes shown in parentheses. (-) shows that no change is shown for the 1978 base year. The ‘Cumulative % Change’ row is the total change from 1978 to 2022.

**Table 6:** Average annual total factor productivity change (%Δ) by region, 1978–2022.

	*n*	TFP	TP	OSEI	EI-FEED	EI-THI	OTEI	SNI
U.S.	1783	1.586	2.523	−0.007	0.023	−0.008	−0.091	−0.833
Midwest	788	1.742	2.523	−0.015	0.035	−0.003	0.005	−0.783
Northeast	478	1.528	2.523	−0.031	0.020	−0.005	−0.092	−0.864
West	397	1.423	2.523	0.049	0.007	−0.021	−0.249	−0.861
South	120	1.332	2.523	−0.043	0.016	−0.001	−0.203	−0.934

## Data Availability

The data presented in this study will be made available by the corresponding author on request. Our study utilizes historical weather data obtained from the Visual Crossing Weather website “https://www.visualcrossing.com/ accessed on 13 February 2024”. This data was acquired through a paid subscription, and the terms of use do not permit us to publicly share or redistribute the raw data.

## Abbreviations

AIC	Akaike Information Criterion
CD	Cobb–Douglas
CEI	Climatic or environmental effect indices
EI-FEED	Environmental index for feed
EI-THI	Environmental index for temperature-humidity
GDD	Growing Degree Days
LR	Log-Likelihood ratio
OSEI	Overall scale efficiency index
OTEI	Overall technical efficiency index
RP	Random parameters
SNI	Statistical Noise Index
SPF	Stochastic Production Frontier
TE	Technical efficiency
TFP	Total factor productivity
TFPG	Total factor productivity growth
TFPI	Total factor productivity index
THI	Temperature-humidity index
TP	Technological progress
TL	Translog

## Appendix A

**Table A1:** Average dairy cow inventory numbers by region and year.

Year	Midwest	Northeast	South	West
1978	2,253,993	1,072,541	248,436	1,099,102
1982	2,465,204	1,116,267	281,961	1,283,054
1987	2,296,207	1,060,823	311,391	1,447,958
1992	2,023,154	972,825	322,738	1,702,456
1997	1,855,120	963,196	315,483	2,013,389
2002	1,711,602	929,936	272,815	2,468,185
2007	1,732,109	874,933	240,150	2,805,179
2012	1,785,092	866,291	205,318	2,845,499
2017	1,812,199	884,323	220,050	2,852,798
2022	1,794,992	825,927	208,585	2,823,267
Avg.	1,972,967	956,706	262,693	2,134,089

Source: Authors’ calculations based on data from the USDA Census of Agriculture.

**Table A2:** Average THI load values by region and year.

Year	Midwest	Northeast	South	West
1978	54.22	36.55	672.24	78.01
1982	46.35	23.96	595.24	88.06
1987	114.44	60.20	570.99	71.50
1992	10.45	9.35	517.39	101.77
1997	47.22	26.66	664.45	93.48
2002	99.00	70.76	668.68	102.33
2007	50.20	29.31	606.46	86.58
2012	98.48	40.37	562.85	87.64
2017	29.35	22.25	639.72	136.85
2022	55.79	45.16	749.08	139.85
Avg.	60.55	36.46	624.71	98.61

Source: Authors’ calculations based on data from VCWD.

**Table A3:** Average GDD values by region and year.

Year	Midwest	Northeast	South	West
1978	2502	2242	4940	3060
1982	2337	2184	4650	3027
1987	2831	2471	4736	3280
1992	2031	2028	4530	3503
1997	2190	2063	4679	3363
2002	2640	2506	4853	3211
2007	2709	2366	4726	3262
2012	2761	2561	4914	3321
2017	2430	2364	4816	3437
2022	2604	2543	5022	3512
Avg.	2504	2333	4787	3298

Source: Authors’ calculations based on data from VCWD.

**Table A4:** Decomposition of average total factor productivity change (%Δ) across U.S. regions and states, 1978–2022.

Region	State	*n*	TFPI	TP	OSEI	EI-FEED	EI-THI	OTEI	SNI
Midwest	Iowa	70	1.457	2.523	−0.013	0.035	0.000	−0.262	−0.804
	Illinois	40	1.585	2.523	−0.070	0.024	−0.007	−0.041	−0.822
	Indiana	20	1.852	2.523	−0.005	0.018	0.003	0.114	−0.784
	Michigan	78	1.893	2.586	0.045	0.023	−0.002	0.055	−0.797
	Minnesota	170	1.657	2.523	−0.039	0.039	0.004	−0.037	−0.813
	Ohio	10	1.714	2.523	−0.003	0.012	0.002	0.011	−0.812
	South Dakota	20	1.926	2.523	0.085	0.061	−0.011	0.054	−0.770
	Wisconsin	380	1.814	2.523	−0.016	0.037	−0.006	0.059	−0.765
Northeast	Connecticut	10	1.288	2.523	−0.062	0.010	−0.013	−0.279	−0.863
	Massachusetts	9	1.749	2.523	−0.052	0.008	0.007	0.036	−0.754
	Maine	10	1.581	2.523	−0.029	0.014	−0.003	−0.065	−0.838
	New Hampshire	30	1.649	2.523	−0.046	0.054	−0.013	−0.038	−0.810
	New York	240	1.689	2.523	−0.019	0.020	−0.005	−0.003	−0.807
	Pennsylvania	69	1.541	2.559	−0.020	0.011	−0.005	−0.110	−0.871
	Vermont	110	1.199	2.523	−0.048	0.021	−0.007	−0.270	−0.991
South	Florida	40	0.577	2.523	−0.065	0.012	−0.006	−0.590	−1.262
	Louisiana	10	1.586	2.523	−0.149	0.013	−0.004	0.020	−0.795
	Maryland	10	1.378	2.523	−0.088	−0.003	0.009	−0.186	−0.852
	Texas	50	1.804	2.523	−0.001	0.026	−0.001	0.018	−0.743
	Virginia	10	1.732	2.523	−0.012	−0.001	0.018	0.012	−0.788
West	Arizona	19	1.485	2.050	0.141	−0.011	−0.007	−0.280	−0.394
	California	149	1.291	2.523	0.022	−0.013	−0.009	−0.328	−0.876
	Colorado	10	1.861	2.523	0.119	0.074	−0.084	0.050	−0.804
	Idaho	80	1.761	2.523	0.114	0.020	−0.031	−0.035	−0.811
	New Mexico	29	1.883	2.208	0.129	0.020	−0.001	0.043	−0.506
	Oregon	20	0.845	2.523	0.016	−0.010	0.007	−0.761	−0.896
	Utah	30	1.365	2.523	0.038	0.016	−0.020	−0.242	−0.923
	Washington	60	1.209	2.523	0.022	0.015	−0.029	−0.385	−0.909
